# An Adaptive Multi-Sensor Data Fusion Method Based on Deep Convolutional Neural Networks for Fault Diagnosis of Planetary Gearbox

**DOI:** 10.3390/s17020414

**Published:** 2017-02-21

**Authors:** Luyang Jing, Taiyong Wang, Ming Zhao, Peng Wang

**Affiliations:** 1 School of Mechanical Engineering, Tianjin University, Tianjin 300354, China; jingluyang@outlook.com (L.J.); pengwang@tju.edu.cn (P.W.); 2 School of Mechanical Engineering, Xi’an Jiaotong University, Xi’an 710049, China; zhaomingxjtu@mail.xjtu.edu.cn

**Keywords:** fault diagnosis, multi-sensor data fusion, deep convolutional neural networks, feature learning, planetary gearbox

## Abstract

A fault diagnosis approach based on multi-sensor data fusion is a promising tool to deal with complicated damage detection problems of mechanical systems. Nevertheless, this approach suffers from two challenges, which are (1) the feature extraction from various types of sensory data and (2) the selection of a suitable fusion level. It is usually difficult to choose an optimal feature or fusion level for a specific fault diagnosis task, and extensive domain expertise and human labor are also highly required during these selections. To address these two challenges, we propose an adaptive multi-sensor data fusion method based on deep convolutional neural networks (DCNN) for fault diagnosis. The proposed method can learn features from raw data and optimize a combination of different fusion levels adaptively to satisfy the requirements of any fault diagnosis task. The proposed method is tested through a planetary gearbox test rig. Handcraft features, manual-selected fusion levels, single sensory data, and two traditional intelligent models, back-propagation neural networks (BPNN) and a support vector machine (SVM), are used as comparisons in the experiment. The results demonstrate that the proposed method is able to detect the conditions of the planetary gearbox effectively with the best diagnosis accuracy among all comparative methods in the experiment.

## 1. Introduction

The planetary gearbox is a key component in mechanical transmission systems and has been widely used in wind turbines, helicopters and other heavy machineries [1]. The wide range of gear ratios, small room in power transmission line, and high transmission efficiency are the most significant advantages of planetary gearboxes [2]. Planetary gearboxes generally operate in tough working environments, which makes them suffer from a variety of failures and damages [1,3] causing unwanted downtime, economic losses, and even human casualties [4]. Therefore, the fault diagnosis of planetary gearboxes is necessary to guarantee a safe and efficient operation of mechanical transmission systems.

Health conditions of planetary gearboxes can be reflected by various kinds of measurements, including vibration signal, acoustic signal, driven motor current, shaft speed, oil debris, etc. Different measurements have different drawbacks and are sensitive to different types of damage modes or operation conditions. Thus, combining and analyzing these measurements together should be an appropriate approach to detect various types of faults of complex systems. This approach, named multi-sensor data fusion, can achieve more accurate and reliable result than approaches with a single sensor [5,6,7].

Nevertheless, multi-sensor data fusion for fault diagnosis suffers from two challenging problems [6]. (1) One is the feature extraction of multi-sensory data. Generally, conventional fault diagnosis includes three steps [8]: signal acquisition, feature extraction, and fault classification. In the feature extraction step, fault sensitive features are extracted and selected from raw data through signal processing technologies and data analysis strategies, such as Fourier spectral analysis, wavelet transformation (WT), and principal component analysis (PCA). However, the multiple types of sensory data of multi-sensor data fusion may cause a number of issues that make the feature extraction with multi-sensor much more difficult than with a single sensor. These issues [6,7] include more imprecision and uncertainties in measurements, various noises, more conflicting or correlating data, higher data dimensions, etc. Extracting features from these kinds of data will be a very challenging and cruel task. At the same time, multiple types of sensory data also increases the difficulties and consumes much time to choose an optimal handcraft feature or manual feature extraction method, even though to date, the optimal handcraft feature or feature extraction method for a specific type of sensory data still remains unanswered [8,9]; (2) The other challenge of the multi-sensor data fusion is the selection of different fusion levels. Similarly, different fusion levels have their own advantages and disadvantages, and the suitable one for different fault diagnosis tasks is usually different [5]. Selecting an optimal fusion level for a specific fault diagnosis task always requires domain expertise, prior knowledge, and human labor.

Deep neural networks (DNN), also known as deep learning, have been attracting increasing attention from researchers from various fields in recent years [10,11,12]. The key advantage of DNN is the feature learning ability [11], which can automatically discover an intricate structure and learn useful features from raw data layer by layer. A number of studies [11,12,13] have shown that DNN can fuse input raw data and extract basic information from it in its lower layers, fuse the basic information into higher representation information and decisions in its middle layers, and further fuse these decisions and information in its higher layers to form the final classification result. It can be seen that DNN itself is a fusion structure [11,13], which fuses the feature extraction, feature selection, data-level fusion, feature-level fusion, decision-level fusion, and classification into one single learning body. For this reason, a DNN-based low level fusion, e.g., data-level fusion, can not only learn features from fused raw data automatically, but also fuse these data, features and decisions adaptively through its deep-layered structure. DNN might be an ideal model to fuse multi-sensory data and detect faults of a mechanical system. However, although some applications [14,15,16,17,18] of DNN in feature learning and fault diagnosis with a single sensor have been found in recent years, no study, to the best of our knowledge, has investigated the effectiveness of DNN-based feature learning and the adaptive level fusion method for fault diagnosis. It is attractive and meaningful to investigate this adaptive fusion method, which can learn features from raw data automatically, and select and combine fusion levels adaptively.

Deep convolutional neural networks (DCNNs), as one of the main types of DNN models, have been successfully used in mechanical fault diagnosis with automatic feature learning from single sensory data [9,14,19,20]. Benefitting from several unique structures, DCNN can achieve better results with less training time than standard neural networks. Firstly, DCNN has a large set of filter kernels in convolutional layers, which can capture representative information and patterns from raw data. Stacking these convolutional layers can further fuse information and build complex patterns; Secondly, DCNN is an unfully-connected network, where each filter shares the same weights. This structure can reduce both the training time and complication of the model. In addition, the pooling layer of DCNN further reduces the revolution of the input data as well as the training time, and improves the robustness of the extracted patterns (a detailed introduction to the DCNN model is presented in Section 2). Thus, DCNN should have great potential in processing the multi-sensory data of a mechanical system, which usually contains rich information in the raw data and is sensitive to training time as well as model size.

Aiming to address the two problems of multi-sensor data fusion mentioned above, this paper proposes an adaptive data fusion method based on DCNN and applies it to detect the health conditions of a planetary gearbox. Different from conventional methods, the proposed method is able to (1) extract features from raw data automatically and (2) optimize a combination of different fusion levels adaptively for any specific fault diagnosis task with less dependence on expert knowledge or human labor.

The rest of the paper is organized as follows. In Section 2, the typical architecture of DCNN and an adaptive training method are briefly described. Section 3 illustrates the procedures of the proposed method, the design of the DCNN model, and several comparative methods introduced to further analyze the performance of the proposed method. In Section 4, an experimental system of a planetary gearbox test rig is used to validate the effectiveness of the proposed method. Finally, the conclusions are drawn in Section 5.

## 2. Deep Convolutional Neural Networks

### 2.1. Architecture of Deep Convolutional Neural Networks

**Figure 1:**
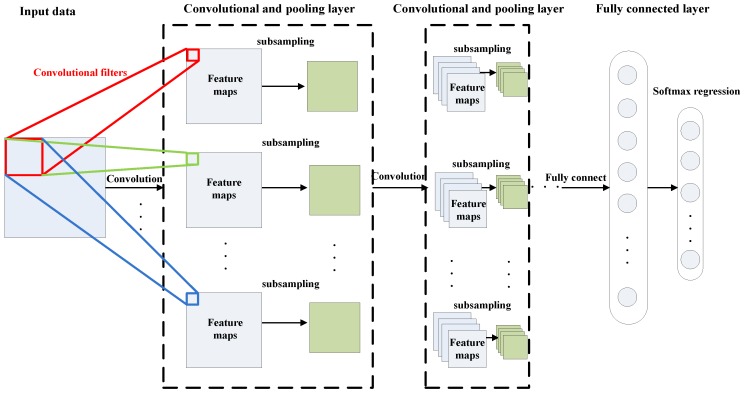
A typical architecture of deep convolutional neural network (DCNN).

DCNN is a type of DNN model inspired by visual system structure [11,21], and it has become the dominant approach for almost all recognition and detection tasks in image and speech analysis [22,23,24]. DCNN contains three kinds of layers [25], which are the convolutional layer, pooling layer, and fully-connected layer. As shown in Figure 1, the first several layers of a typical DCNN usually consist of a combination of two types of layers—convolutional layers, followed by pooling layers—and the last layer is a fully-connected layer. In the following part, we will describe these three kinds of layers in more detail.

The convolutional layer is composed of a number of two-dimensional (2D) filters with weighted parameters. These filters convolute with input data and obtain an output, named as feature maps. Each filter shares the same weighted parameters for all the patches of the input data to reduce the training time and complication of the model, which is different from a traditional neural network with different weighted parameters for different patches of the input data. Suppose the input of the convolutional layer is X, which belongs to $R^{A\times B}$, and *A* and *B* are the dimensions of the input data. Then the output of the convolutional layer can be calculated as follows [26]:

(1) $$C_{cn}=f\left(\overset{CC}{\underset{cc=1}{\sum }}X_{cc}^{l-1}*W_{cn}^{l}+B_{cn}^{l}\right)$$

where $C_{cn}$ is the *cn*-th output of the convolutional layer, and the output number is *CN*, which is also equal to the filter number; * is an operator of convolution; $X_{cc}$ represents the input data of *cc-*th channel of previous layer l − 1, and the channel number is *CC*; $W_{cn}^{l}$ is the weight of *cn*-th filter of the current layer *l*; the width and height of the filter are *CW* and *CH*, respectively; the *cn*-th bias is denoted with $b_{cn}^{l}$; f is an activation function, typically hyperbolic tangent or sigmoid function.

The pooling layer is a sub-sampling layer, which reduces the revolution of the input data and improves the robustness of learned features. A pooling layer generally follows a convolutional layer with a max pooling method and it outputs only the maximum of each sub-sampling patch of the feature maps to subsample the feature maps from the previous convolutional layer. The output can be described as follows [26]:

(2) $$P_{cn}=\underset{C_{cn}\in S}{\max}C_{cn}$$

where $P_{cn}$ is the *cn*-th output of the pooling layer, and the output number is *CN*; *S* is the pooling block size. This function will sum over each distinct *S* pooling block in the input data so that the output will become *S* times smaller along both spatial dimensions.

The fully-connected layer is the last layer of the DCNN model. It follows several combinations of the convolutional layers and the pooling layers, and classifies the higher-level information from the previous layers. A fully-connected layer is similar to a traditional multilayer neural network with a hidden layer and a classification layer, typically using a softmax regression. Assuming that the task is a *K*-label problem, the output of the softmax regression can be calculated as follows:

(3) $$O_{j}=\left[\begin{array}{c} P(y=1|x;\theta )\\ P(y=2|x;\theta )\\ \begin{array}{c} \ldots \\ P(y=k|x;\theta ) \end{array} \end{array}\right]=\frac{1}{\sum_{j=1}^{K}\exp\left(\theta^{\left(j\right)}x\right)}\left[\begin{array}{c} \exp\left(\theta^{\left(1\right)}x\right)\\ \exp\left(\theta^{\left(2\right)}x\right)\\ \begin{array}{c} \ldots \\ \exp\left(\theta^{\left(K\right)}x\right) \end{array} \end{array}\right]$$

where $\theta^{\left(1\right)},\theta^{\left(2\right)},\ldots \theta^{\left(K\right)}$ are the parameters of the model, and $O_{j}$ is the final result of the DCNN.

### 2.2. Training Method

It can be seen from the previous description that $w_{i}^{l}$, $b_{cn}^{l}$, and $\theta^{\left(j\right)}$ are the learnable parameters and will be optimized through model training with gradient decent algorithms. Since a gradient decent algorithm is easily trapped into local optima, we introduce several enhancement methods, including stochastic gradient decent (SGD), cross-validation, and adaptive learning rate, to solve this problem. SGD updates gradient [27] based on a few training data instead of the entire training set. This approach not only increases the training speed, but also improves the training reliability. Cross-validation selects a validation set from training data to test the performance of the parameters of the model to avoid overfitting. Since a global constant learning rate easily causes either a slow convergence with a lower learning rate or a serious fluctuation of the convergence with a higher learning rate, an adaptive learning rate is employed. The adaptive learning rate has a high rate at first and decreases with the increase of the training epochs adaptively to obtain a fast and reliable convergence result.

## 3. Adaptive Multi-Sensor Data Fusion Method Based on DCNN for Fault Diagnosis

### 3.1. Procedure of the Proposed Method

An adaptive multi-sensor data fusion method based on DCNN is presented to learn features from raw data automatically and combine fusion levels adaptively to detect faults of a planetary gearbox. Through its deep-layered structure, DCNN can fuse input data and extract basic features in the lower layers, fuse basic features into high level features and decisions in the middle layers, and further fuse these features and decisions in the higher layers to obtain the final diagnosis result.

**Figure 2:**
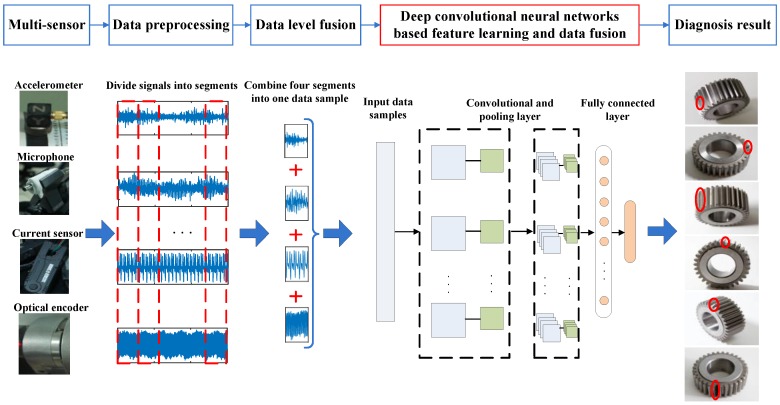
Flowchart of the proposed method.

Figure 2 displays the flowchart of the proposed method: (1) four types of signals, including vibration signal [3,28,29], acoustic signal [4,30], current signal [31,32,33], and instantaneous angular speed (IAS) signal [34,35], are selected according to published studies and acquired from a planetary gearbox; (2) data preprocessing is applied to standardize each signal and divide them into segments; (3) each of the four segments of the four signal types are combined together simply as one data sample to form the data-level fused input data of the DCNN model; (4) DCNN is trained and tested with these fused input data, and its output will be the diagnosis result of the planetary gearbox. The testing accuracy of the output result is used to evaluate the effectiveness of the proposed method. It should be noted that although we use a data-level fusion in the third step, data is fused again in the starting layers of the DCNN model to further optimize the data structure. The DCNN implicitly contains data-level fusion, feature-level fusion, and decision-level fusion through the deep-layered structure and it optimizes a combination of these fusion levels adaptively according to the characteristic of the data itself.

### 3.2. Model Design of DCNN

The model of DCNN is adjusted to satisfy the characteristics of mechanical fault diagnosis. Although most applications of DCNN in image recognition chose a 2D convolutional structure [11,22,36], and some researchers [20,37] also used the same way to diagnose mechanical faults, we select a one-dimensional (1D) convolutional structure with a 1D filter bank as the kernel of the DCNN model. In our opinion, the main reason behind the applications of the 2D convolutional structure of DCNN in image analysis lies in the natural 2D space correlation in images. However, most of the measurement data for mechanical fault diagnosis only correlate with time, which is a 1D parameter. Thus, 1D convolutional structure should be an appropriate choice for a DCNN-based fault diagnosis problem. In addition, we choose a larger filter size than conventional ones used in image recognition. While a larger size of the filter may be more expensive in terms of computation, a larger filter can capture more information between the data farther away from each other [38], which may be the features in the frequency domain.

In spite of the many benefits of the deep-layered structure of DCNN, a “deep” structure also means complicated hyper-parameters as well as various choices of architectures, which increases the difficulty to build an appropriate and efficient model. Although there are several researches [39,40] about the automatic optimization of parameters of DCNN, the optimized process is usually time-consuming and easily converges into a local optimum due to the large number of parameters of DCNN. Thus, we build the DCNN model initially based on a few general design principles [38,41]. Then several configurations of the network are tested using the experimental data, and the one with best performance is selected as the setting of the final model.

### 3.3. Comparative Methods

**Figure 3:**
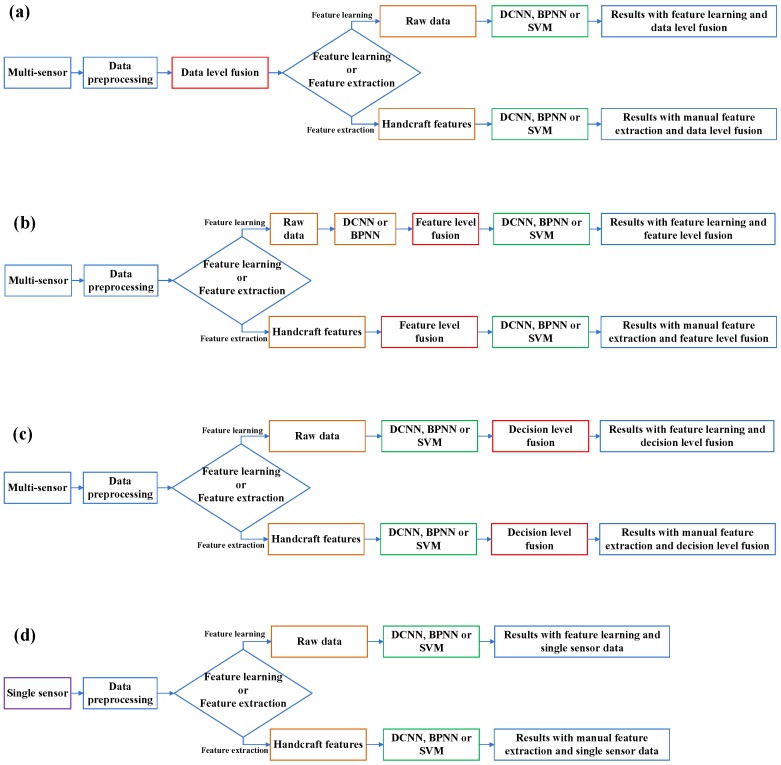
Flowcharts of comparative methods: (**a**) Data-level fusion methods; (**b**) Feature-level fusion methods; (**c**) Decision-level fusion methods; and (**d**) Methods with single sensory data.

Several comparative methods are employed to further test and confirm the performance of the proposed method. The flowcharts of comparative methods are shown in Figure 3.

To evaluate the ability of learning features from the raw data of the proposed method, manual feature extraction is used as a replacement and comparison of the feature learning in each comparative method. Eight time-domain features and several frequency-domain features are extracted. Root mean square (RMS), kurtosis, crest factor, skewness, mean, minimum, maximum, and variance are chosen as the handcraft features in the time domain [34,37,42]. The characteristic frequencies of the planetary gearbox [1], including the rotating frequencies of the sun gear, planetary gear and the carrier, the pass frequency of the planetary gear and the meshing frequency of the planetary gearbox, are selected as the handcraft features in the frequency domain as well as their ten sidebands for all types of the sensor signals [34,43,44] except for the current signal. For the current signal, the line frequency and its sidebands [45] generated by the modulation of the characteristic frequencies of the planetary gearbox are chosen as its frequency-domain features. In addition, the fast Fourier-transform (FFT) energy of each sample, which is obtained by splitting the frequency spectrum of each sample into 32 average bands and calculating the RMS of each band [46], is also added into the handcraft features in the frequency domain. While all the domain features are processed together as the “handcraft features”, the “time-domain features” and “frequency-domain features” are also tested respectively to reflect more information about the sensitivity of the data. The comparison between the learning features from raw data and the handcraft features is marked in orange in Figure 3.

As a comparison of the DCNN model of the proposed method, two intelligent models, back-propagation neural networks (BPNN) and support vector machine (SVM), are introduced as replacements of DCNN in each comparative method. BPNN is built into a three-layer model with sigmoid activation functions. SVM uses Gaussian radial basis function (RBF) as the kernel function and the grid search method to optimize the parameters of the kernel. The three comparative models, DCNN, BPNN, and SVM, are marked in green in Figure 3.

For testing the performance of the different fusion levels, manual-selected feature-level fusion and decision-level fusion are explored and compared with the data-level fusion of the proposed method. For feature-level fusion with feature learning from raw data, only DCNN and BPNN are applied to learn features from the raw data of each sensor, respectively. The outputs of the second-last layers of DCNN are extracted as the learned features of each sensory data. Then, all the learned features from the four types of sensors are combined together as the feature-level fused features and used as the input of another DCNN for classification. In the same way, the outputs of the second-last layers of BPNN are extracted and fused. Then, the fused features are used as the input of both BPNN and SVM for classification. The comparison of different fusion levels is marked in red in Figure 3.

As a comparison of the multi-sensory input data of the proposed method, fault diagnosis with single sensory data is also applied to evaluate the effectiveness of the proposed method, which is marked in purple and shown in Figure 3d.

## 4. Experiment and Discussion

### 4.1. Experiment Setup

**Figure 4:**
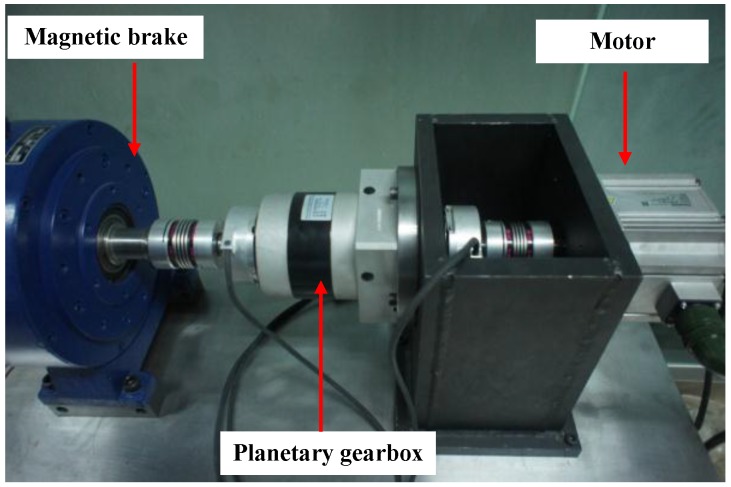
Planetary gearbox test rig.

An experimental system of a planetary gearbox test rig is established to evaluate the effectiveness of the proposed method. As shown in Figure 4, it consists of a one-stage planetary gearbox, a driven motor and a magnetic brake. The planetary gearbox contains one 20-tooth sun gear and three 31-tooth planetary gears, and transmits torque from the sun gear to the planetary carrier of the planetary gears with a standstill ring gear.

**Figure 5:**
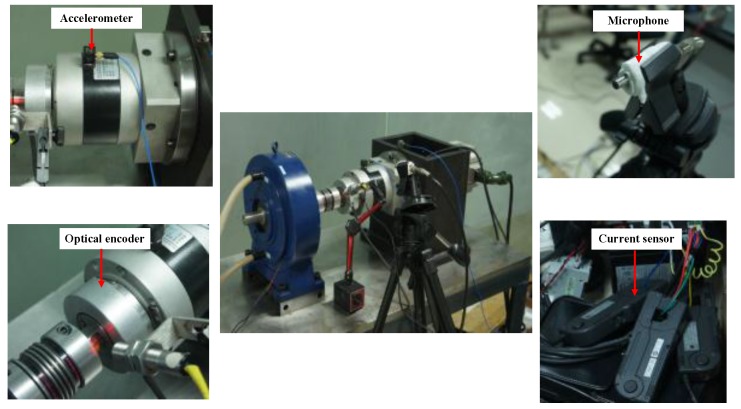
Four types of sensors and their installations.

Figure 5 presents the four types of sensors employed in the experiment, including an accelerometer, microphone, current sensor, and optical encoder. Vibration signal, acoustic signal, and motor current signal are measured by their corresponding sensors and acquired through a data acquisition box with a sampling frequency of 20 kHz and data length of 320 k points. The IAS of the output shaft is calculated based on counting the number of high resolution pulses of the encoder [47]; and down-sampling, using the data acquisition box with the same sampling frequency and data length as the other three signals.

**Figure 6:**
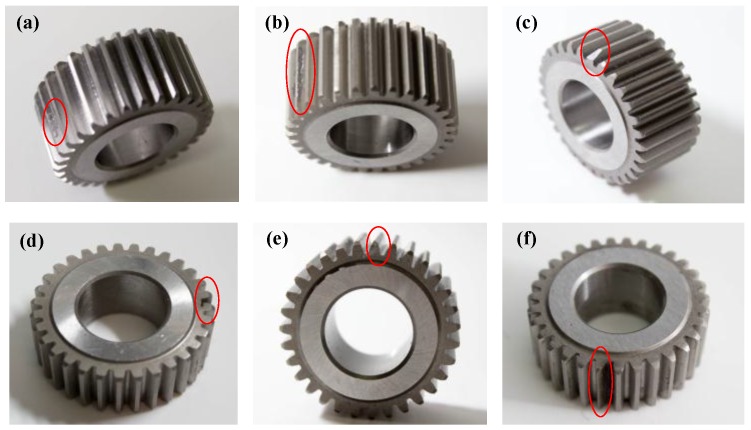
Faulty planetary gears: (**a**) Pitting tooth; (**b**) Chaffing tooth; (**c**) Chipped tooth; (**d**) Root crack tooth; (**e**) Slight worn tooth; and (**f**) Worn tooth.

Seven health conditions of the planetary gearbox are tested, including normal, pitting tooth, chaffing tooth, chipped tooth, root crack tooth, slight worn tooth, and worn tooth. As shown in Figure 6, all the faults occurred in the planetary gears. In each experiment, only one planetary gear with one kind of health condition is examined, and all the other gears are normal. Experiments are conducted under three motor speeds (600 rpm, 1200 rpm and 1800 rpm) and a load-free condition. The detailed description for the datasets and pattern labels of the experiment is shown in Table 1.

### 4.2. Data Processing

The acquired vibration signal, acoustic signal, current signal, and IAS signal are preprocessed following the steps in Section 3.1. For the proposed method, the collected signals are standardized and divided into segments at first. A total of 1024 points are selected as a segment, so each type of signal will contain 312 segments for each health condition under one motor speed and 6552 segments in total for seven health conditions under three motor speeds. Next, each of the four segments of the four signal types are combined together as one data sample to form the input vectors of the DCNN model. In this way, each data sample will be a 4096-dimensional vector (four times the size of segments), and there will be 6552 data samples in total. A total of 40% of the 6552 data samples are selected randomly as the training data set, 10% are used as the validation set, and the remaining 50% are selected as the testing data set. Eight trails are carried out to avoid particularity and contingency of the diagnosis result. The average testing accuracy of the eight trails is calculated as the final result.

### 4.3. Model Design

The model of the DCNN is developed using the principles described in Section 3.2. For different input data, different model settings are tested, and the one with the best performance among all the tested settings is selected to process this input data. The model setting of the proposed method is displayed in Table 2, which consists of three convolutional layers, two pooling layers, and a fully-connected layer with softmax regression. The convolutional layer corresponds to Equation (1), the pooling layer to Equation (2) and the fully-connected layer to Equation (3). The DCNN model is developed based on C++.

### 4.4. Experimental Results

#### 4.4.1. Results of Single Sensory Data

Following the flowchart shown in Figure 3d in Section 3.3, methods with three intelligent models, feature learning and manual feature extraction methods, and four types of single sensor data are tested in the experiment. The results are displayed in Table 3.

#### 4.4.2. Results of Multi-Sensory Data

**Figure 7:**
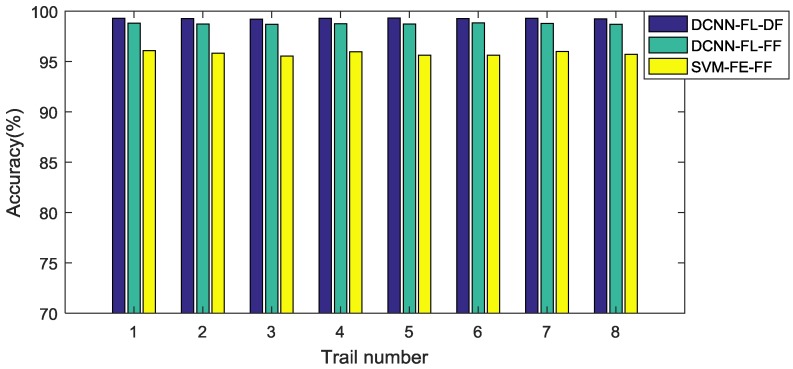
Testing accuracy of eight trials of the top three methods (the proposed method, the DCNN model with feature learning and feature-level fusion, and the SVM model with handcraft features and feature-level fusion). FL = feature learning; FE = manual feature extraction; DF = data-level fusion; FF = feature-level fusion.

Following the flowcharts shown in Figure 3a–c in Section 3.3, methods with three fusion levels, three intelligent models, two feature extraction methods, and multi-sensory data are tested in the experiment. The results are displayed in Table 4, in which the result of the proposed method is marked in bold. Figure 7 presents the testing results of the eight trails of the top three methods, which are the proposed method, the DCNN model with feature learning and feature-level fusion, and the SVM model with handcraft features and feature-level fusion.

**Figure 8:**
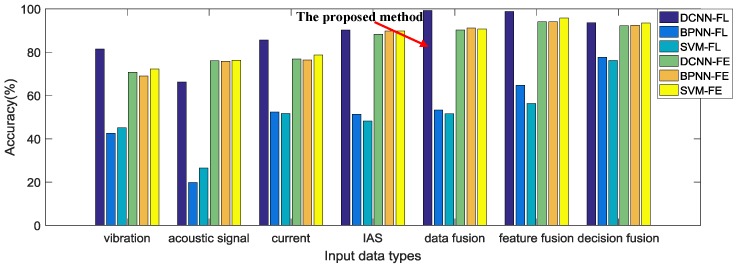
Testing accuracy of all the comparative methods.

Finally, the average testing accuracies of all the comparative methods in the experiment are shown together in Figure 8 for a clearer comparison between each other.

### 4.5. Principal Component Analysis of the Experimental Data and Learned Features

**Figure 9:**
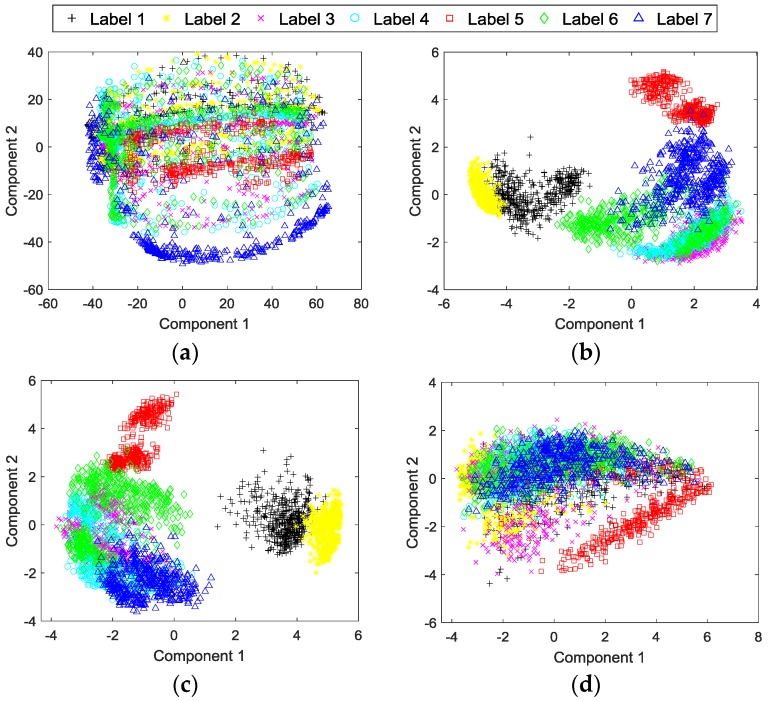
Principal component analysis (PCA) of the experimental data and learned features: (**a**) Data-level fused input data of the proposed method; (**b**) Features of the proposed method; (**c**) Feature-level fused features learned through DCNN; and (**d**) Feature-level fused handcraft features.

PCA is employed to analyze and visualize the learned features of the proposed method. As shown in Figure 9, the labels 1 to 7 correspond to the seven conditions of the planetary gearbox described in Table 1, and the first two principal components (PCs) are obtained by PCA to represent the useful information hidden in the data. Figure 9a shows the result of the input data of the testing dataset of the proposed method along the first two PCs. Figure 9b illustrates the result of the learned features with adaptive fusion levels of the proposed method for testing the dataset, which are extracted from the output of the second-last layer of the DCNN. For comparison, the results of feature-level fused features learned through DCNN and feature-level fused handcraft features along the first two PCs are shown in Figure 9c,d, respectively. It should be noted that we only display the first two PCs of the data for a clearer visualization, which means that there may be overlaps between some categories and many of them can be divided into higher PCs.

### 4.6. Discussion

1. The experimental results show that the proposed method is able to diagnose the faults of the planetary gearbox test rig effectively, yielding the best testing accuracy in the experiment. It can be seen from Table 4 and Figure 8 that the proposed method achieves the best testing accuracy 99.28% among all the comparative methods. We think that this result is significantly correlated with the deep architecture of the DCNN model of the proposed method. DCNN can fuse input data and learn basic features from it in its lower layers, fuse basic features into higher level features or decisions in its middle layers, and further fuse these features and decisions to obtain the final result in its higher layers. Although there is a data-level fusion before DCNN in the proposed method, DCNN still actually fuses the data again in its starting layers to further optimize the data structure. Optimized features and combinations of different level fusions are formed through this deep-layered model, which provides a better result than with manually selected features or fusion levels.
2. The ability of automatic feature learning of the DCNN model with multi-sensory data is proven through the experiment. It can obviously be seen from Figure 8 that both the proposed method and the feature-level fusion method with feature learning through DCNN obtain a better result, 99.28% and 98.75%, than any other comparative methods with handcraft features or feature learning through BPNN. This result proves that the feature learning through DCNN with multi-sensory data can improve the performance of the multi-sensor data fusion method for fault diagnosis. In addition, the result also implies that the proposed method with adaptive fusion-level selection can achieve a better result 99.28% than the result 98.75% of the method with manual-selected feature-level fusion, which is the only difference between these two methods.
3. However, the method with automatic feature learning of DCNN from the raw signal of a single sensor cannot achieve a better result than methods with handcraft features. Table 3 displays the diagnosis results using signals from a single sensor. Only with a vibration signal and current signal, can the DCNN-based feature learning method achieve better results than conventional methods with handcraft features. By contrast, the results of the DCNN-based feature learning method with an acoustic signal and IAS signal are worse than that of conventional methods. This implies that the DCNN-based method with learned features from single sensory data cannot provide stable improvements for all kinds of sensory data. We think that the performance of the DCNN-based feature learning is influenced by the characteristics of the input data. As can be seen from the results shown in Table 3, the performance of feature learning has a stronger positive correlation with the performance of time-domain features than frequency-domain features, which infers that the DCNN-based feature learning from a raw signal may be more sensitive to time-correlated features than frequency-correlated features.
4. The effectiveness of the automatic feature learning and adaptive fusion-level selection of the proposed method is further confirmed through PCA. As can be seen from Figure 9a, most of the categories of the input raw data overlap each other, which makes it difficult to distinguish them. After the processing of the proposed method, the learned features with adaptive fusion levels along the first two PCs become distinguishable in Figure 9b. Meanwhile, Figure 9c,d presents the results of PCA with feature-level fused learned features and handcraft features as comparisons, respectively. The feature-level fused features learned through DCNN have just a slightly worse distinction between each category than the features of the proposed method, which not only verifies the feature learning ability of DCNN used in both methods, but also proves the better performance of the adaptive-level fusion of the proposed method than that of the manual-selected feature-level fusion. On the contrary, the fused handcraft features show a much worse distinction between different categories than the learned features of the proposed method. These analyses further demonstrate the effective performance of the automatic feature learning and adaptive fusion-level selection of the proposed method.
5. While DCNN has a much better feature learning ability than BPNN, the three comparative models, DCNN, BPNN and SVM, obtain similar results with handcraft features. Figure 8 shows clearly that feature learning through DCNN achieves much better testing accuracies than through BPNN. Nevertheless, with handcrafts features, these three intelligent models provide similar accuracies, which suggests that DCNN cannot achieve much more improvements than conventional methods without using its ability of feature learning.
6. Methods with multi-sensory data provide better results than those with single sensory data. It can be seen from Figure 8 that methods with multi-sensory data achieve higher testing accuracies than with single sensory data, no matter which fusion level or intelligent model is selected. This phenomenon indicates that multi-sensory data can improve the reliability and accuracy for fault diagnosis.

## 5. Conclusions and Future Work

This paper presents an adaptive data fusion method based on DCNN to detect the health conditions of planetary gearboxes. The processes of data-level fusion, feature-level fusion, decision-level fusion, feature learning, and fault diagnosis are all fused into one DCNN model adaptively. The proposed method can learn features from raw data, and fuse data, features, and decisions adaptively through the deep-layered structure of DCNN with fewer requirements of expert knowledge or human labor for feature extraction and fusion-level selection. The performance of the proposed method is evaluated through the experiment of the planetary gearbox fault test rig. As comparisons, feature-level fusion, decision-level fusion, handcraft features, single sensory data, and two traditional intelligent models, BPNN and SVM, are also tested in the experiment. The comparative results of the experiment verify the effectiveness of the proposed method, which achieves the best testing accuracy among all the comparative methods in the experiment.

Our future work will focus on testing the DCNN model-based feature learning and data fusion approaches on more mechanical objects, fault modes, operation conditions, and sensor types, which can further confirm the effectiveness of approaches and help us to find out other useful application guidance. Moreover, due to the large number of parameters of deep learning models, manual parameter optimization often takes many trials-and-errors to find the best one, and conventional automatic searching methods are usually very time-consuming and easily converge into a local optimum. It is meaningful to investigate more effective and faster approaches to optimize the parameters automatically. Finally, combinations of different deep learning architectures should improve the effect of fault diagnosis. Adding recurrent architecture may make the model suitable to predict future fault conditions, and combining with auto-encoder architecture may improve the feature learning ability to capture more complex features.

## Figures and Tables

**Table 1:** Description of the pattern labels of planetary gearbox data.

Pattern Label	Gearbox Condition	Input Speed (rpm)	Load
1	Normal	600, 1200 and 1800	Zero
2	Pitting tooth	600, 1200 and 1800	Zero
3	Chaffing tooth	600, 1200 and 1800	Zero
4	Chipped tooth	600, 1200 and 1800	Zero
5	Root cracked tooth	600, 1200 and 1800	Zero
6	Slight worn tooth	600, 1200 and 1800	Zero
7	Worn tooth	600, 1200 and 1800	Zero

**Table 2:** Variables of parameters and structures of the deep convolutional neural networks.

Layer	Type	Variables and Dimensions	Training Parameters
1	Convolution	CW = 65; CH = 1; CC = 1; CN = 10; B = 10	SGD minibatch size = 20
2	Pooling	S = 2	Initial learning rate = 0.05
3	Convolution	CW = 65; CH = 1; CC = 10; CN = 15; B = 15	Decrease of learning rate after each ten epochs = 20%
4	Pooling	S = 2	Momentum = 0.5
5	Convolution	CW = 976; CH = 1; CC = 15; CN = 30; B = 30	Weight decay = 0.04
6	Hidden layer	Relu activation function	Max epochs = 200
7	Softmax	7 outputs	Testing sample rate = 50%

SDG = stochastic gradient decent; CW = filter width; CH = filter height; CC = filter channel; CN = number of filter in the bank; B = bias; S = sub-sampling rate; No overlapping of convolutional window and no padding.

**Table 3:** Average testing accuracy of comparative methods with single sensory data.

Sensory Data	Model	Feature Learning from Raw Data	Manual Feature Extraction		
			Time-Domain Features	Frequency-Domain Features	Handcraft Features
Vibration signal	DCNN	81.45%	55.84%	70.74%	73.64%
	BPNN	42.56%	55.62%	69.03%	72.36%
	SVM	45.11%	56.35%	72.23%	73.86%
Acoustic signal	DCNN	66.23%	31.42%	76.45%	76.02%
	BPNN	19.80%	35.89%	76.04%	75.79%
	SVM	26.54%	33.62%	77.36%	76.32%
Current signal	DCNN	85.68%	60.73%	61.45%	76.85%
	BPNN	52.36%	60.47%	61.21%	76.43%
	SVM	51.64%	63.74%	63.53%	78.76%
Instantaneous angular speed (IAS) signal	DCNN	90.23%	75.34%	84.42%	88.34%
	BPNN	51.37%	75.36%	85.22%	89.82%
	SVM	48.22%	75.68%	85.65%	89.85%

DCNN = deep convolutional neural network; BPNN = back-propagation neural networks; SVM = support vector machine.

**Table 4:** Average testing accuracy of comparative methods with multi-sensory data.

Fusion Level	Model	Feature Learning from Raw Data	Manual Feature Extraction		
			Time-Domain Features	Frequency-Domain Features	Handcraft Features
Data-level fusion	DCNN	99.28%	66.08%	87.63%	90.23%
	BPNN	53.28%	65.95%	87.89%	91.22%
	SVM	51.62%	67.32%	87.28%	90.67%
Feature-level fusion	DCNN	98.75%	86.35%	92.34%	94.08%
	BPNN	64.74%	86.81%	92.15%	94.04%
	SVM	56.27%	86.74%	94.62%	95.80%
Decision-level fusion	DCNN	93.65%	84.65%	90.23%	92.19%
	BPNN	77.62%	84.47%	91.19%	93.42%
	SVM	76.17%	86.32%	90.98%	93.44%
